# ORA BIOMEDICAL: ACCELERATING DRUG DISCOVERY FOR GEROSCIENCE

**DOI:** 10.1093/geroni/igae098.2298

**Published:** 2024-12-31

**Authors:** Michael Muir, Benjamin Blue, Matt Kaeberlein, Mitchell Lee

**Affiliations:** Ora Biomedical, Inc., Seattle, Washington, United States; Ora Biomedical, Inc., Seattle, Washington, United States; Optispan, Inc., Seattle, Washington, United States; Ora Biomedical, Inc., Seattle, Washington, United States

## Abstract

Small molecule interventions that directly target the molecular mechanisms of aging have the potential to revolutionize 21st century health and preventative medicine. Altering the trajectory of aging holds incredible economic value in humans, companion pets, and other animals. The longevity biotechnology sector is quickly growing to meet this opportunity, but a relatively small number of interventions and aging targets are poised for clinical development. Ora Biomedical, Inc. is a pharmaceutical company launched out of the Kaeberlein Lab at the University of Washington. We routinely test natural products, pharmaceuticals, combinatorial mixtures, and biologics to identify those that impact biological aging. Our drug screening uses the WormBot-AI platform, which leverages AI, robotics, and C. elegans to enhance the efficiency and accuracy of pre-clinical drug discovery. The WormBot-AI is the best in class for high-throughput intervention testing with lifespan, disease progression, and health measures as primary endpoints. We have also used our platform to launch the Million Molecule Challenge (MMC), a crowdfunded open science initiative. We show here the open access data crowd-funded by scientists and community members from over twenty countries. Furthermore, as a resource and tool for the broader aging science community, we have launched the MMC dashboard. This allows any member of the aging research community to access this crowd-funded data. Ora Biomedical is currently soliciting partnerships with both academic and non-academic aging researchers. We offer a wide array of screening services in multiple disease and mutant models as well as custom intervention validation experiments.

